# Deregulated Phosphorylation of CENH3 at Ser65 Affects the Development of Floral Meristems in *Arabidopsis thaliana*

**DOI:** 10.3389/fpls.2019.00928

**Published:** 2019-07-25

**Authors:** Dmitri Demidov, Stefan Heckmann, Oda Weiss, Twan Rutten, Eva Dvořák Tomaštíková, Markus Kuhlmann, Patrick Scholl, Celia Maria Municio, Inna Lermontova, Andreas Houben

**Affiliations:** ^1^Leibniz Institute of Plant Genetics and Crop Plant Research, Gatersleben, Germany; ^2^Centre of Plant Structural and Functional Genomics, Institute of Experimental Botany Academy of Sciences, Olomouc, Czechia; ^3^Department of Plant Biology, Uppsala BioCenter and Linnean Center for Plant Biology, Swedish University of Agricultural Sciences, Uppsala, Sweden; ^4^Independent Researcher, Plankstadt, Germany

**Keywords:** CENH3, phosphorylation, Aurora kinase, floral meristem, *Arabidopsis*

## Abstract

Several histone variants are posttranslationally phosphorylated. Little is known about phosphorylation of the centromere-specific histone 3 (CENH3) variant in plants. We show that CENH3 of *Arabidopsis thaliana* is phosphorylated *in vitro* by Aurora3, predominantly at serine 65. Interaction of Aurora3 and CENH3 was found by immunoprecipitation (IP) in *A. thaliana* and by bimolecular fluorescence complementation. Western blotting with an anti-CENH3 pS65 antibody showed that CENH3 pS65 is more abundant in flower buds than elsewhere in the plant. Substitution of serine 65 by either alanine or aspartic acid resulted in a range of phenotypic abnormalities, especially in reproductive tissues. We conclude that Aurora3 phosphorylates CENH3 at S65 and that this post-translational modification is required for the proper development of the floral meristem.

## Introduction

Histone3 (H3) is the best studied histone variant, regarding its post-translational modifications (PTMs). In the centromeric region of most eukaryotic chromosomes, H3 is replaced by CENH3, originally referred to as CENPA in human (Earnshaw and Rothfield, 1985), or as HTR12 in *Arabidopsis thaliana* (Talbert et al., 2002). The incorporation of CENH3 into centromeric nucleosomes initiates the formation of the kinetochore, a protein complex which enables the microtubules to attach to the centromere (Earnshaw et al., 2013). CENH3 features a well conserved histone fold domain and a highly variable N-terminus. Non-plant CENH3s experience a variety of PTMs. For example, the trimethylation of glycine 1, along with the phosphorylation of serine 16, and serine 18 has been observed in cultured human cells (Bailey et al., 2013; Takada et al., 2017). Of yeast CENH3, the arginine residue 37 can be methylated, serine at position 9, 10, 14, 16, 17, 22, 33, 40, 105, and 154 are phosphorylated, and lysine 49 is acetylated (Samel et al., 2012; Boeckmann et al., 2013; Hoffmann et al., 2018; Mishra et al., 2019). The human CENPA serine 7 is phosphorylated during mitosis by the cell cycle-dependent Aurora kinase (Zeitlin et al., 2001; Kunitoku et al., 2003), an enzyme which can also phosphorylate histone H3 at serine 10 and 28 (Hsu et al., 2000; Kurihara et al., 2006), and the H1 serine residue 27 (Hergeth et al., 2011).

Phosphorylation of CENH3 is likely required for kinetochore function and normal chromosome segregation (Boeckmann et al., 2013; Goutte-Gattat et al., 2013). Phosphorylation of CENH3 at S68 by the cyclin-dependent kinase 1 prevents interaction with the chaperone HJURP which is required for loading of CENH3 to centromeric nucleosomes (Yu et al., 2015; Wang et al., 2017). The only documented PTM involving a plant CENH3 is phosphorylation of the maize CENH3 pS50, which has been interpreted as a spindle assembly checkpoint (Zhang et al., 2005). The protein kinase responsible for this phosphorylation has not yet been identified.

Plant Aurora kinases have been classified in two major subgroups, referred to as α and β type Aurora (Demidov et al., 2005; Kawabe et al., 2005; Kato et al., 2011). The *A. thaliana* genome encodes two α (AtAurora1 and 2) and one β (Aurora3) type Aurora kinases. These kinases are concentrated at the centromeres, and in the phragmoplast at the end of the mitotic cell division. Alignment of plant Auroras with the animal Aurora A and B types (Adams et al., 2001) revealed characteristics of both animal enzyme classes as well as plant-specific features (Demidov et al., 2005). Aurora3 phosphorylates the serine residues 10 and 28 of *A. thaliana* H3 (Kurihara et al., 2006).

Here, we aimed to elucidate whether *A. thaliana* CENH3 is phosphorylated by Aurora3. We show that CENH3 is a substrate of Aurora3 and that serine 65 of CENH3 is phosphorylated preferentially in meristematic tissues such as flower buds and flowers. Additionally, we demonstrate that CENH3 pS65 is important for the proper development of reproductive tissues and how the disturbance of CENH3 phosphorylation can in addition impair the growth and development of the whole plant.

## Materials and Methods

### Plant Growth and Transformation

Ecotype Columbia-0 and heterozygous *cenh3-1/CENH3* (Ravi and Chan, 2010) *A. thaliana* plants were transformed using the floral dip method (Clough and Bent, 1998). T1 transformants were selected on Murashige and Skoog solid medium containing the relevant antibiotic(s) and were grown under either a 16 h or an 8 h photoperiod with a day/night temperature regime of 20°C/18°C. *Nicotiana benthamiana* and *Nicotiana tabacum* plants were grown under a 12 h photoperiod at a constant temperature of 26°C.

### DNA Extraction and Genotyping

Genomic DNA was extracted according to Edwards et al. (1991). Selection for the *cenh3-1* allele was achieved using a dCAPS marker: the template was amplified using the primer air cenh3-1_mut_for/_rev and the amplicon digested with *Eco*RV. The amplicon of the 215 bp mutant allele is resistant to digestion, while the Wt allele splits into a 191 bp, and a 24 bp fragments. To distinguish between the endogenous CENH3 copy and the two CENH3 transgenes carrying S65A or S65D, an initial PCR based on the primer pair cenh3-1_mut_for/_mut2429r was performed: the amplicon was then used as a template for a second PCR/dCAPS assay as described above. Primer sequences are given in Supplementary Table S1.

### Cloning of Transformation Constructs

To generate CENH3 genomic fragments carrying either S65A or S65D, a genomic fragment of CENH3 with its native 1500 bp-long promotor, inserted in the plasmid pCAMBIA1300 was excised by *Hin*dIII/*Bam*HI digestion, and then cloned into pBlueScript II KS (Stratagene). The S65A and S65D mutations were generated in the cloned copy using a Phusion^®^ site-directed mutagenesis kit (Finnzymes^1^) according to supplier’s protocol; the required 5′-phosphorylated primers were S65_A_for, S65_D_for and S65_A+D_gDNA_rev. The *CENH3* S65A and S65D sequences were excised by *Hin*dIII/*Bam*HI digestion and re-inserted. To generate the *CENH3* S65A and S65D fusions to EYFP, the *p35S:CENH3YFP* expression cassette (Lermontova et al., 2006) was processed using a Phusion^®^ site-directed mutagenesis kit (Finnzymes): the required primers were S65_A_for, S65_D_for and S65_A+D_cDNA_rev. The expression cassettes (*p35S, CENH3YFP*-S65, -A65 or -D65 and *NOS* terminator) were restricted with *Sfi*I and inserted into the pLH7000 vector^2^. All constructs were verified by sequencing. Primer sequences are given in Supplementary Table S1.

### Heterologous Expression in *E. coli*

Full length *CENH3* and Aurora3 cDNAs were amplified using a RevertAid H minus first strand cDNA synthesis kit (Thermo Fisher Scientific^3^), and inserted, after removal of the stop codon, into pENTR-D TOPO (Thermo Fisher Scientific, see text footnote 4). The *CENH3* open reading frame sequence was amplified using the primer pair CENH3_expr_for/_rev with Phusion High-Fidelity DNA Polymerase (Fermentas), and the amplicon inserted into a Champion^TM^ pET101 Directional TOPO^®^ Expression plasmid (Invitrogen). The sequence of the *CENH3* variant carrying the S65A substitution was obtained amplifying the wild-type plasmid with a mutated primer with a Phusion^®^ site-directed mutagenesis kit (Finnzymes) using the primer S65_A_for. The constructs were transformed into *E. coli* BL21 (GE Healthcare Life Sciences^4^). The fragment of Aurora3 obtained by PCR based on the primer pair Aurora3G-LP/-RP was inserted into pDest15 (Invitrogen) to create a GST fusion tag and then transformed into *E. coli* C43 (Lucigen^5^). An active Aurora3 kinase was synthesized in *E. coli* as described by Swain et al. (2008). The synthesis of recombinant protein was induced by addition of 1 mM IPTG to the *E. coli* culture during the exponential growth phase. CENH3 and CENH3 S65A 6xHIS-fusions were purified by passing through a Ni-NTA agarose column (Qiagen^6^) under denaturing conditions, and dialyzed against urea (Tomaštíková et al., 2015). Recombinant proteins were tested by Coomassie staining of Tris–glycine (Laemmli, 1970) or Tris–tricine (Schagger and von Jagow, 1987) PAAGE. Primer sequences are given in Supplementary Table S1.

### *In vitro* Kinase Assay

The *in vitro* protein kinase assays of recombinant Aurora3 and the CENH3 variants were performed as described by Karimi-Ashtiyani and Houben (2013). *In vitro* protein kinase assays of recombinant Aurora3 on CENH3 peptides were performed as described by Demidov et al. (2009). The required CENH3 peptides were synthesized by JPT Peptide Technologies GmbH^7^. Each *in vitro* kinase reaction was based on 2 μg peptide.

### Bimolecular Fluorescence Complementation (BiFC) Constructs and the Detection of Fluorescence

An Aurora3 pENTR-D TOPO construct was generated using the Gateway system (Invitrogen, see text footnote 4) within the binary BiFC plant transformation vectors pSpyce-35S and pSpyne-35S (Walter et al., 2004). Leaves of 2–4 week old either *N. benthamiana* or *N. tabacum* plants were infiltrated on their abaxial side with the *Agrobacterium tumefaciens* strain C58C1 carrying the pCH32 helper plasmid. The p19 protein of tomato bushy stunt virus was used to suppress gene silencing (Walter et al., 2004). Co-infiltration was performed with equal titers of *A. tumefaciens* containing either a BiFC construct or the p19 silencing plasmid. The fluorescent protein fusion constructs used as controls for the localization of Aurora3 and CENH3 were previously described (Demidov et al., 2005; Lermontova et al., 2006). The preparation of tissue for confocal fluorescence microscopy followed the methods described by Keçeli et al. (2017). YFP was detected by a LSM780 laser scanning microscope (Carl Zeiss, Jena, Germany) using a 488 nm laser line for excitation in combination with a 505–550 nm band pass for detection (Lermontova et al., 2013). Specificity of the YFP signal was confirmed by photospectrometric analysis of the fluorescence signal by means of the META detector.

### Analysis of Total Plant Protein

Plant tissue (200–300 mg) was powdered in liquid nitrogen and suspended in 0.5 mL 56 mM Na2CO3, 56 mM DTT, 2% w/v SDS, 12% w/v sucrose, and 2 mM EDTA. After holding for 10 min at 70°C, the cell debris was removed by centrifugation. A 30 μg aliquot of each total protein sample was analyzed by 10% PAGE containing acetic acid-urea (Spiker, 1980) or 10% Tris–tricine PAGE (Schagger and von Jagow, 1987) and either stained in Coomassie Blue or electro-transferred onto Immobilon TM PVDF membranes (Millipore^8^). The membranes were challenged with either anti-*A. thaliana* CENH3 (Abcam^9^) or a polyclonal anti-CENH3 pS65 antibody (antibodies were working only for Western blotting but not for indirect immunostaining) produced by Pineda Antibody Service (Berlin, Germany). The latter was raised against a synthetic phosphorylated pS65 residue (± 10 AA) peptide and was purified from serum using immobilized peptides (1, with pS65 residue and 2, with S65 residue). The specificity of the antibody was validated using an ELISA. The membranes were held for 12 h at 4°C in PBS containing 5% w/v low-fat milk powder and a 1:1,000 dilution of polyclonal rabbit anti-CENH3 or anti-CENH3 pS65 and monoclonal mouse H3 (Abcam, see text footnote 10). Bound antibodies were detected by incubation with anti-rabbit or anti-mouse antibodies conjugated to peroxidase (Sigma^10^) in a dilution 1:5,000 and visualized using an enhanced chemiluminescence assay (Pierce, see text footnote 4).

### λ Phosphatase Treatment

Aliquots of ∼100 μg protein were incubated for 1 h at 30°C in 100 μL of a pH 7.5 buffer containing 50 mM HEPES, 100 mM NaCl, 2mM DTT, 1 mM MnCl2, 0.01% v/v Brij-35 in the presence of 100 U alkaline phosphatase (Sigma), and 4,000 U λ phosphatase (NEB^11^).

### Immunoprecipitation (IP)

The protein preparations required for IP were extracted from 10-day old transgenic seedlings harboring the pCENH3:CENH3:YFP construct in *cenh3-1* mutant plants after crosslinking with dithiobis succinimidyl propionate (DSP) for 30 min and 4°C according to the supplier’s (Thermo Fisher Scientific^TM^ Pierce^TM^) protocol. A 10 g sample of plant material was powdered in liquid nitrogen, and then extracted in 50 mL 200 mM Tris–HCl (pH7.5), 1.5 M NaCl, and 0.5% v/v Tween20. After 20 min centrifugation at 4°C and 15000 *g*, the supernatant was diluted with dH_2_O 1:10 and incubated with 12 μL GFP-Trap resin (ChromoTek GmbH^12^) for 4 h at 4°C. The GFP-Trap agarose was rinsed in 1×PBS and eluted according to the supplier’s protocol. A 15 μL aliquot of each protein sample was electrophoretically separated and transferred to a membrane which was then probed with an anti-AtAurora antibody (Demidov et al., 2005). An extract of wild type (Wt) seedling was used as negative control.

### Indirect Immunofluorescence

Immunostaining was performed as described by Manzanero et al. (2000). Rabbit antibodies raised against Nicotiana CENPC (Nagaki et al., 2009) and a mouse antibody raised against GFP (Clontech^13^) were diluted 1:400 in PBS. The binding of the primary antibody was detected by using as a 1:200 diluted secondary antibody of either rhodamine-conjugated anti-rabbit IgG (Dianova^14^) or FITC-conjugated anti-mouse IgG (Dianova).

### Preparation of Pollen Mother Cells for Meiotic Analysis

Flower buds were fixed and pollen mother cells prepared for microscopy following Sánchez-Morán et al. (2001) with minor modifications. The buds were fixed in Carnoy’s solution (absolute ethanol:chloroform:glacial acetic acid, 6:3:1), rinsed first in 3:1 ethanol:glacial acetic acid, and then in citrate buffer (pH 4.5) at room temperature. They were then softened by digestion in 0.3% w/v pectolyase, 0.3% w/v cytohelicase, and 0.3% w/v cellulase (Sigma) in citrate buffer for 2 h at 37°C. The enzyme mixture was replaced by an ice-cold citrate buffer to stop the reaction. Single buds were transferred onto a clean microscope slide in a drop of citrate buffer and macerated with a fine needle. A 10 μL aliquot of 60% (v/v) glacial acetic acid was added to each slide, which was then dried by laying on a hotplate (42°C) for 1 min, after which again 10 μL 60% (v/v) acetic acid was added, followed by 200 μL of cold 3:1 ethanol:glacial acetic acid. Finally, the slides were air-dried.

### Scanning Electron and Light Microscopy

The preparation of tissue sections for scanning electron and light microscopy followed the methods described by Tikhenko et al. (2015).

### Alexander Staining

Flowers and flower buds were immersed in 10% ethanol overnight at 10°C, and the anthers were stained following Alexander (1969) for 15 min at room temperature. Estimates of pollen grain numbers per anther were based on the inspection of six anthers per plant.

### Accession Numbers

The following line was used: *cenh3-1/CENH3* (At1g01370, Ravi and Chan, 2010).

## Results

### CENH3 Interacts With AtAurora Kinase *in vivo*

**FIGURE 1:**
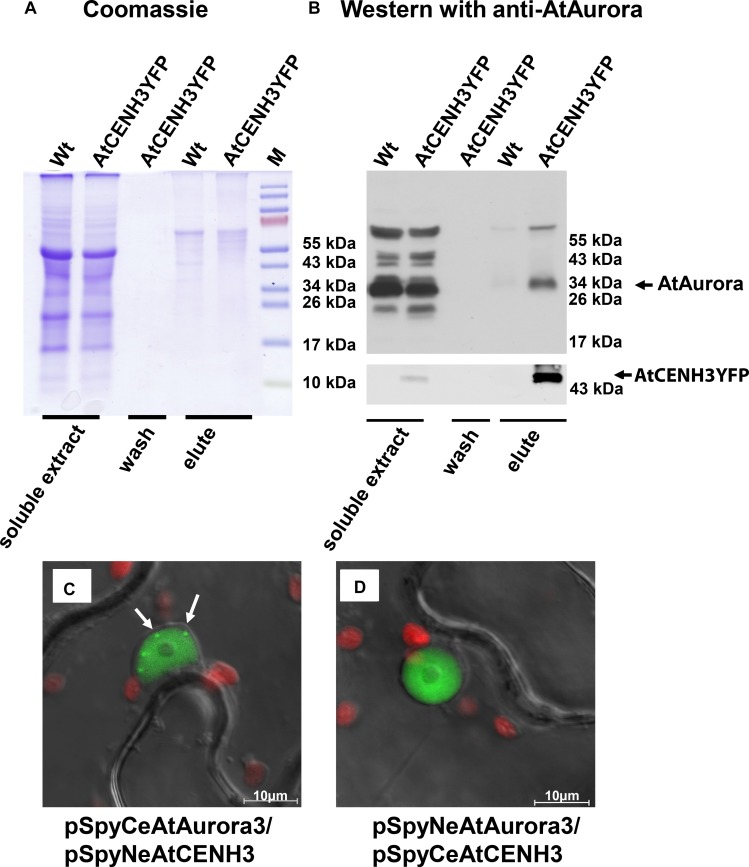
*Arabidopsis thaliana* CENH3 interacts with Aurora3 kinase. **(A,B)** Western blot analysis using immuno-precipitated samples of *A. thaliana CENH3YFP* transformants and Wt plants. **(A)** Proteins separated by gel electrophoresis and visualized by Coomassie Blue staining. **(B)** Western blot probed with an anti-AtAurora, or anti-YFP (bottom part) antibodies. The negative control consisted of proteins extracted from Wt plants. **(C,D)** *In vivo* interaction of Aurora3 with CENH3 in the nucleoplasm as shown by BiFC in *N. benthamiana* leaves infiltrated with *A. tumefaciens* harboring the constructs **(C)** pSpyCeAurora3/pSpyNeCENH3 and **(D)** pSpyNeAurora3/pSpyCeCENH3. Some centromeres in **(C)** are arrowed. Red fluorescence corresponds to the autofluorescence of chlorophyll in the plastids.

The *in vivo* interaction between *Arabidopsis* Aurora and CENH3 was investigated using immunoprecipitation (IP) and bimolecular fluorescence complementation (BiFC). The former experiment was based on soluble protein isolated from DSP cross-linked *A. thaliana* seedlings stably expressing CENH3YFP (Lermontova et al., 2006). Western blot analysis of the precipitates eluted from anti-GFP agarose (Figures 1A,B) revealed that a ∼34 kDa product which interacted with Aurora was more abundant in CENH3YFP expressing plants than in the wild type (Wt) controls (Figure 1B). The 65–70 kDa product observed could have been either a dimerized form of Aurora and/or a complex formed with other proteins and stabilized by crosslinking. The corresponding negative control with YFP only does not show signals in the elution fraction (Supplementary Figure S1). In the validating BiFC experiment, YFP-specific signals were detected within the nucleoplasm of infiltrated *N. benthamiana* cells (Figures 1C,D). A number of pSpyNeAurora3/pSpyCeCENH3 spot-like BiFC signals were observed in the nucleus of infiltrated leaves (Figure 1C, arrowed). The distribution of the BiFC signal differed from that of Aurora3GFP, which was concentrated in the nucleoplasm and around the cell periphery (Supplementary Figure S2A), but was comparable with the distribution of CENH3YFP (Supplementary Figure S2B). Similarly, distinct CENH3YFP fluorescence signals were generated when nuclei isolated from *N. tabacum* cells transiently expressing CENH3YFP were labeled with antibodies against both YFP and CENPC (Supplementary Figure S2K). None of the negative controls produced a fluorescence signal (Supplementary Figures S2C–J). These results suggest that CENH3 interacts with Aurora3 *in planta*.

### Aurora3 Phosphorylates CENH3 *in vitro* at the Serine 65 Residue

**FIGURE 2:**
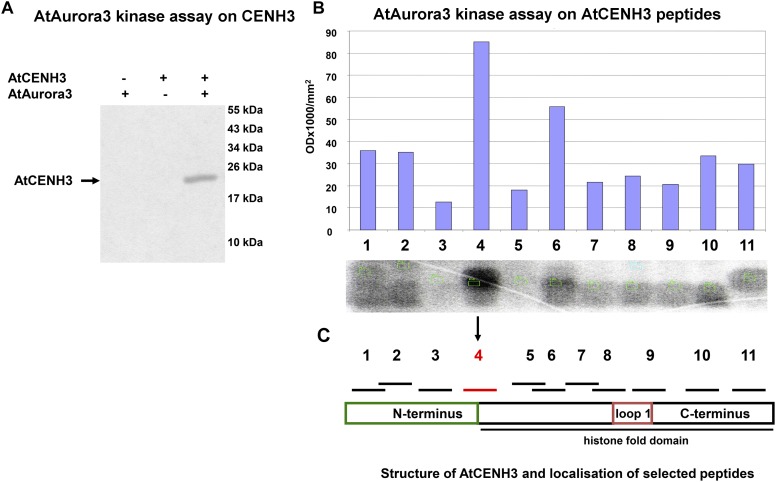
Aurora3 phosphorylates CENH3 *in vitro*. **(A)** A kinase assay using recombinant CENH3 as substrate. Kinase reactions either without substrate or with no added enzyme were used as negative controls (–). **(B)** *In vitro* kinase screening of CENH3 phosphorylation sites based on 11 synthetic peptides containing putative Aurora kinase recognition motifs as substrate. **(C)** Schematic localization of selected peptides on CENH3.

To assess whether Aurora3 can phosphorylate CENH3 *in vitro*, recombinant Aurora3 and CENH3 were produced in *E. coli*. Presenting recombinant CENH3 (Supplementary Figure S3A) as the substrate for recombinant active Aurora3 (Supplementary Figure S3B) resulted in the production of kinase activity-dependent Western blot signals (Figure 2A), thus indicating phosphorylation of CENH3. The phosphorylation sites were identified by scanning CENH3 for the putative Aurora kinase phosphorylation motifs (R/K)1-3X(S/T) based on the information available for non-plant Aurora kinases (Dephoure et al., 2008; Gully et al., 2012). The resulting eleven putative target sites (Supplementary Figure S3D), each embedded within an 11–20 residue oligopeptide (Supplementary Figure S3E), were then tested as a potential substrate of Aurora3. Screening of preselected peptides by the *in vitro* kinase assay showed the strongest phosphorylation signal when the serine 65 residue was present (Figure 2B). This residue locates at the border between the N- terminus and the histone fold domain (Figure 2C). The Aurora3-dependent phosphorylation of the S65 residue was confirmed by showing that an S65A variant of CENH3 (Supplementary Figure S3C), which cannot undergo phosphorylation at this position, was phosphorylated with twofold lower efficiency than Wt CENH3 (Supplementary Figures S4A,B).

### CENH3 Phosphorylation at Serine 65 Is Abundant in Reproductive Tissue

**FIGURE 3:**
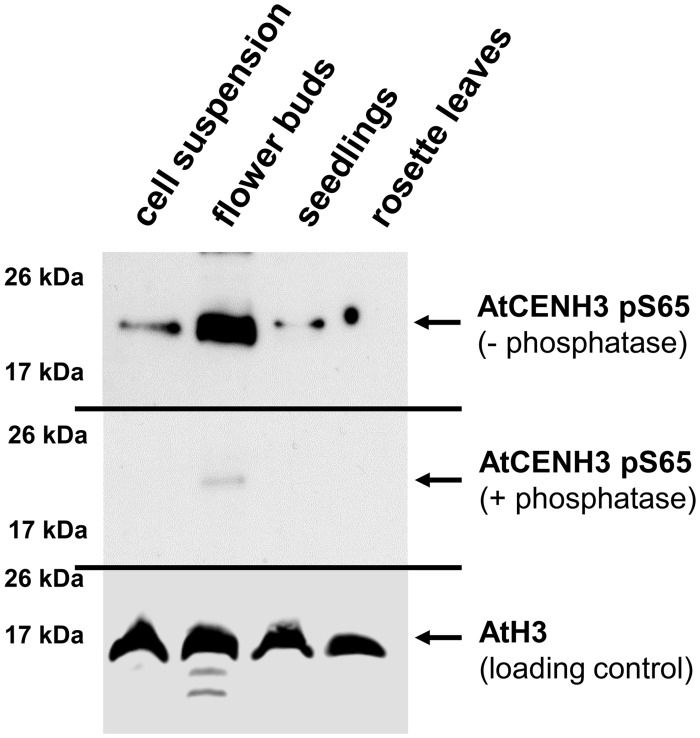
CENH3 is phosphorylated at serine 65 in tissues with a high frequency of cell divisions. Western blot analysis, based on an anti-CENH3 pS65 antibody, shows variation between samples isolated from different *A. thaliana* tissues. Upper panel, non-treated samples; central panel: samples treated with phosphatase; lower panel, loading control (anti-histone H3). Total CENH3 amount and Coomassie staining for the same samples are shown in Supplementary Figure S4C.

To analyze the presence and abundance of the CENH3 pS65 *in planta*, polyclonal antibodies were raised against the AtCENH3 peptide phosphorylated at serine 65 (hereafter CENH3 pS65). On Western blot performed, the strongest anti-CENH3 pS65 signal was detected in extracts of flower buds and the weakest in mature leaves (Figure 3). Western blotting with the same protein samples after phosphatase treatment showed only a weak immune signal, thereby demonstrating that the antibodies are specific to the phosphorylated form of AtCENH3, and retained only residual antigenicity to the non-phosphorylated form (Figure 3). A parallel Western blot using anti-CENH3 revealed a rather different distribution of signals (Supplementary Figure S4C). Here, strongest signals were found in proteins isolated from a rapidly growing cell suspension, and there was no quantitative difference in intensity between λ phosphatase-treated and non-treated samples. Although the quantity of CENH3 of the cell suspension extract was greater than that present of the flower bud extract (Supplementary Figure S4C), the quantity of phosphorylated CENH3 was greater in the flower buds (Figure 3), suggesting a possible physiological function of CENH3 phosphorylation during the development of reproductive tissue.

### CENH3 Phosphorylation at Serine 65 Is Involved in Development of Vegetative and Especially Reproductive Tissues

**FIGURE 4:**
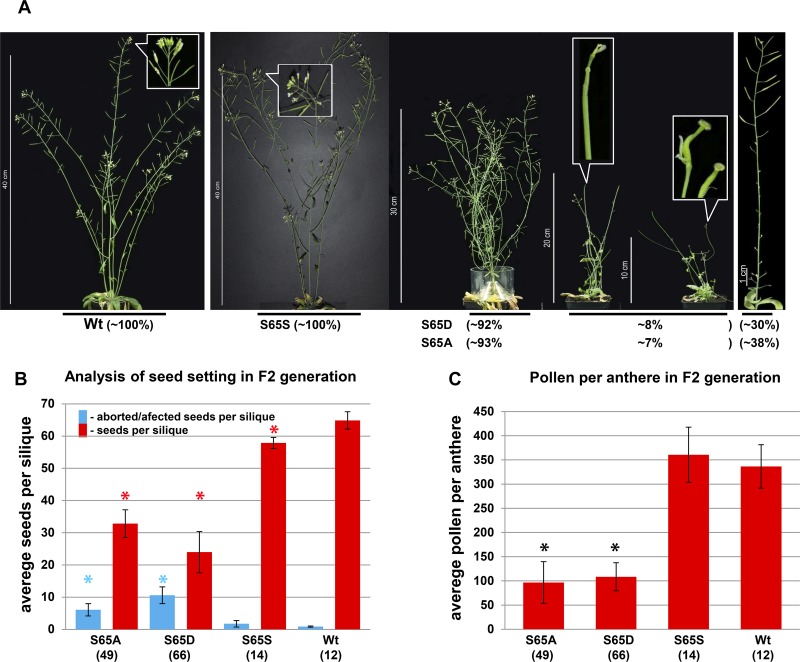
Replacement of serine 65 of CENH3 results in an abnormal plant growth and flower development. **(A)** Complementation of the *cenh3-1* mutant with transgenes encoding either the CENH3 variants: Wt, S65D and S65A under native promotor, results in various phenotypical abnormalities especially involving reproductive structures (plants at 60 DAS). For each construct, 12 to 78 independent transgenic lines were obtained. **(B)** The CENH3 S65A and S65D transgenes do not rescue the *cenh3-1* mutant with respect to seed set as fully as does transgenic Wt CENH3. The average number of seeds per silique was determined based on seven siliques per plant. The numbers shown in parentheses refer to the number of independent lines used for analysis. Error bars correspond to standard deviation. **(C)** The complementation of *cenh3-1* by CENH3 S65A or S65D does not fully restore male fertility. The number of pollen per anther was determined based on fifteen anthers per plant. The average numbers shown in parentheses refer to the number of independent lines used for analysis. Error bars correspond to standard deviation. Columns indicated with asterisks were significantly different in comparison with Wt. The data were analyzed by one-way ANOVA-test (^*^p < 0.05). The calculations were performed with the statistical program SigmaPlot v12 (Systat Software, Inc.).

**FIGURE 5:**
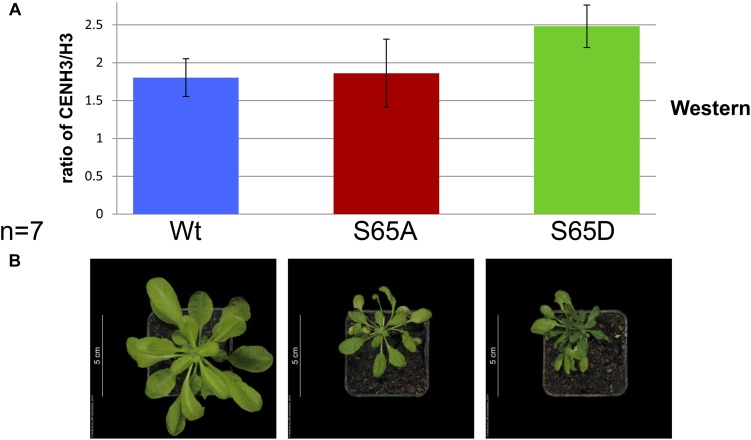
The phenotype of *cenh3-1* mutants harboring *CENH3* S65A or S65D does not correlate with the ratio of CENH3: H3. **(A)** The average ratio of CENH3 to H3 (based on a Western blot using LI-COR Image Studio 3.1 analytic software) in Wt and *cenh3-1* plants harboring *CENH3* S65A or S65D. The experiment was performed in seven biological replicates with protein extracted from 7 × 5 independent transgenic F2 plants and 7 × 5 Wt plants. Error bars correspond to standard deviation. **(B)** Shows typical phenotype of plants (20 DAS) analyzed in **(A)**.

To study in more detail the role of CENH3 pS65, we generated cDNA or genomic CENH3 constructs containing S65A, and S65D mutations. To visualize the sub-cellular localization of S65 phosphorylated CENH3, YFP fusion constructs containing CENH3 cDNA either with the S65A or the S65D mutation under the 35S promoter were transformed into wild-type *A. thaliana* plants. *In vivo* fluorescence analysis of plants expressing the CENH3 S65A or the S65D variant revealed a concentration of YFP signal at the centromeres, which was not distinguishable from the distribution seen in control plants – CENH3YFP (Supplementary Figures S5A–C). To understand the functional role of S65 phosphorylated CENH3, heterozygote *cenh3-1* knock-out mutant plants were transformed with S65A, or S65D mutated CENH3 genomic constructs under the endogenous promoter. We assumed that the S65A mutation would prevent phosphorylation of CENH3 at this residue whereas the S65D mutation imitates the steric configuration and negative charge of phosphorylated S65 (Eot-Houllier et al., 2018). Both variants were able to rescue the lethality associated with homozygosity for the *cenh3-1* mutation. A Western blot analysis based on an antibody recognizing CENH3 pS65 confirmed that neither of the two complemented lines experienced phosphorylation at position 65 (Supplementary Figures S6A–C). Phenotype analysis of the generated lines revealed no visible alteration in the growth of seedling roots (Supplementary Figure S11). A moderate difference in the vegetative growth rate at early growing stages (Supplementary Figures S7, S10) especially between 10 and 50 days after seeding (DAS), (compared to either the *cenh3-1* mutants transformed with CENH3 S65S or with Wt) was observed for 92% of the S65D-complemented and 93% of the S65A-complemented plants (Figure 4A and Supplementary Table S3). About 8% of the S65D-complemented and 7% of the S65A-complemented plants showed a stronger reduction in the growth rate and impaired development of floral meristems and reproductive organs (Figure 4A and Supplementary Figure S8). The affected plants formed no anthers or petals, although their pistil appeared to be normal (Figure 4A and Supplementary Figures S9A,B). In general, *cenh3-1* mutants complemented by S65A or S65D showed the same number (Supplementary Table S3) but a reduced biomass of leaves (Supplementary Figure S10) and were later flowering than Wt and S65S complemented lines (Supplementary Figure S7 and Supplementary Table S2). It should be noted that in older plants (∼63 DAS), *cenh3-1* complemented mutants (S65A or S65D) showed an increased number of lateral stems compared to Wt, and S65S complemented lines (Supplementary Figure S7 and Supplementary Table S3). Around 30% of the S65D-complemented and 38% of the S65A-complemented plants were largely sterile and only their most distal siliques set a reduced number of seeds compared to Wt or *cenh3-1* mutants transformed with CENH3 S65S (Figures 4A,B). Even the sum of developed and aborted seeds is lower in *cenh3-1* complemented mutant plants (S65A or S65D) than in Wt and S65S complemented lines. In addition, the number of viable pollen grains in anthers of *cenh3-1* complemented mutants (S65A or S65D) was reduced compared to Wt and S65S complemented plants (Figure 4C and Supplementary Figure S12). Further, the male meiosis (130 pollen mother cells) of S65 mutant plants did not show any obvious abnormality compared to Wt (*n* = 250), nor was there any evidence for aneuploids or polyploids among their progeny. Western blot analysis with anti-CENH3 antibodies of protein extracts from complemented and Wt plants showed despite clear growth differences (20 DAS), a similar abundance of CENH3 in all plants (Figures 5A,B). *In silico* expression analysis of Aurora3 and CENH3 in different tissues revealed the highest expression levels of both genes in floral and shoot apical meristems (Supplementary Figures S13A,B).

## Discussion

### AtAurora3 Interacts With CENH3 *in vivo* and Phosphorylates It *in vitro*

We have shown that Aurora3, likely an *A. thaliana* ortholog of human Aurora B, interacts with AtCENH3 *in vivo* and phosphorylates it *in vitro*. In human cells, both Aurora A and B interact and phosphorylate CENH3 (Kunitoku et al., 2003; Slattery et al., 2008), but the interaction is stronger with Aurora B (Yu et al., 2015). In *A. thaliana*, Aurora3 is associated with the centromeres during mitosis (Demidov et al., 2005). Here IP revealed *Arabidopsis* Aurora kinase binding with AtCENH3 in extracts of growing seedlings after crosslinking with DSP, which retained *in vivo* protein-protein interactions in the presence of 1.5 M NaCl in the extraction buffer. A high salt concentration is needed to release CENH3 from nucleosomes. Additionally, BiFC demonstrated the interaction between Aurora3 and AtCENH3 *in vivo*, specifically at the centromeres after infiltration in tobacco leaves. This finding is in accordance with the observation that heterologous CENH3 may localize to the centromeres in addition to the endogenous variant of CENH3 (see review by Lermontova and Schubert, 2013). *In vitro* phosphorylation of CENH3 by Aurora kinase has been demonstrated in a range of organisms (Kunitoku et al., 2003; Slattery et al., 2008). Since the CENH3 N-terminal sequence is highly variable (even between closely related species), the determination of phosphorylation sites used by Aurora3 in *A. thaliana* was not feasible based on sequence homology. Nevertheless, the Aurora recognition motif, which was experimentally determined closely resembled that used by Aurora A and Aurora B (Dephoure et al., 2008; Gully et al., 2012). Nine of the 11 peptides carrying Aurora recognition sites were weakly phosphorylated, suggesting that multiple CENH3 phosphorylation sites are targeted in *A. thaliana*. These data were confirmed by the kinase assay using recombinant CENH3 as substrate. Additionally, phosphorylation of CENH3 by Aurora3 *in vitro* also demonstrates the interaction of both proteins. Substitution of serine 65 reduced but did not fully abolish CENH3 phosphorylation (Supplementary Figures S4A,B) indicating the existence of multiple putative AtAurora3 phosphorylation sites. The sequence context of CENH3 S65 was found in most, but not all Brassicaceae genomes (Maheshwari et al., 2015). However, at position threonine 65 occurs more often than serine (Supplementary Figure S14A). In some cases, even a non-phosphorylatable alanine residue is present, irrespective of whether the species encodes one or multiple CENH3 variants (Supplementary Figure S14B). Other conserved blocks within the N-terminal region of plant CENH3s also harbor Aurora recognition motifs, though at a lower frequency. Possibly, conserved sequence blocks could be favored recognition motifs for enzymes involved in post-translational CENH3 modifications. The CENH3 N-terminus can also be phosphorylated by other kinases, for example, by the cyclin-dependent kinase 1 (Yu et al., 2015). The cell cycle-dependent phosphorylation of maize CENH3 at S50 (Zhang et al., 2005) is likely not mediated by Aurora kinase because of the lack of a recognition motif for this enzyme in the vicinity of this site.

The use of a CENH3 pS65-specific antibody confirmed the identity of the phosphorylation sites identified *in vitro* and *in vivo* since binding was abolished after the protein sample had been phosphatase-treated. In agreement with Heckmann et al. (2011), who showed that the intensity of CENH3 expression is related to the extent of cell division occurring in a certain tissue, the present analysis indicated that the abundance of CENH3 was particularly high in actively dividing cells, e.g., in suspension cultures. However, phosphorylation of CENH3 at S65 was particularly high in protein extracts from flower buds. Phosphorylation of CENH3 S65 is not restricted to generative tissue since it also occurred in seedlings and in suspension cultures; and it does not strictly correlate with cell division, because its occurrence was relatively low in actively dividing suspension cultures. Assuming a tissue type-dependent regulation of CENH3 phosphorylation would be consistent with the tissue type-dependent organization and regulation of centromeres (Kagawa et al., 2014; Ishii et al., 2015).

### Phosphorylation of CENH3 S65 by Aurora3 Is Required for Proper Development of Floral Meristems

In order to uncover the physiological role of S65 phosphorylation *in vivo*, *cenh3-1* mutant plants were transformed with CENH3 gene variants displaying either S65A or S65D. Since the expression of Aurora3 and CENH3 depends on the cell cycle (Demidov et al., 2005; Heckmann et al., 2011; Lermontova et al., 2011a) and both are invovled in cell cycle control (Schumacher et al., 1998; Howman et al., 2000), we assume that the phosphorylation of serine 65 is cell cycle dependent in some meristematic tissues. This assumption is in line with the observation that in 30-days-old *cenh3-1* mutants complemented by S65A or S65D the average size of epidermal cells, is smaller than that of Wt or of S65S complemented lines (Supplementary Figure S15A). In 50-days-old plants, this difference is abolished (Supplementary Figure S15B), and the size of leaf epidermal cells and plant age seem to be correlated (Kalve et al., 2014). Some of the S65A- and S65D-complemented *cenh3-1* mutant plants displayed defective differentiation of the apical meristem and developed shoots without male reproductive structures (Figure 4A and Supplementary Figures S8, S9). We assume that in *cenh3-1* complemented mutants (S65A or S65D), defects in the early floral meristem development lead to slower cell growth, and reduced biomass. This effect disappeared during later stages (Supplementary Figure S7 and Supplementary Table S2). After 63 DAS, plants remain smaller compared to the controls (Wt and S65S). CENH3 transgenics (S65A and S65D) showed an increased number of lateral stems (Supplementary Table S3). We speculate that, over time, the blocking of shoot meristem development in S65 mutants is released. Partial compensation of such blocking is visibly by an increase in the number of lateral stems. However, they are smaller than in control plants because they already lost the time for normal growth during the life cycle. Therefore, CENH3 transformants (S65A and S65D) generally remain slightly smaller in size compared to controls (WT and S65S).

Because Aurora3 and CENH3 are strongly expressed in the floral meristem, phosphorylation mutations of CENH3 might contribute to the observed morphological changes of male generative organs. Most likely, the dynamic CENH3 phosphorylation of serine 65 is involved in the regulation of floral meristem development, as implied by the high abundance of pS65 in flower buds (Figure 3), and by the phenotypic effect of S65 substitutions which resembled those seen in plants expressing CENH3 variants with a modified N-terminus (Ravi et al., 2011) or just the CENH3 histone fold domain (Lermontova et al., 2011b).

Interestingly, overexpression of AtAurora1 in tobacco similarly results in an altered stamen morphology, in a reduced growth rate and in enlarged axillary meristems (Demidov et al., 2014). Down-regulation of *Arabidopsis* Aurora1 and Aurora3 results in reduced biomass, slow development, reduced pollen fertility and seed setting (Demidov et al., 2014). This phenotype is reminiscent, but not completely identical to, that of the S65A- and S65D-complemented *cenh3-1* mutants.

Two reasons why no phenotypic differences between the S65A- and the S65D-complemented *cenh3-1* mutants were found can be envisaged. Either the S65D complementation does not functionally compensate for S65 phosphorylation, in spite of its steric similarity to pS65. Alternatively, S65 undergoes highly dynamic phosphorylation and dephosphorylation, and both substitutions (S65A and S65D) impair the functionality of CENH3 and therefore result in the observed phenotype. Comparable results were observed for tissue-cultured human CENH3 mutants (Zeitlin et al., 2001). Substitution of CENH3 Ser7 by Ala or Glu (Glu like Asp imitates pSer) equally leads to an increase of the Flemming body lifetime and midbody length in comparison with the control. The authors explained this observation by a possible change in the CENH3 N-termini structure or due to the disruption of the phosphorylation/dephosphorylation dynamics of Ala7 and Glu7 mutated CENH3.

For a better understanding of the mechanisms involved in CENH3 S65 phosphorylation and its physiological significance in the development of meristems, additional experiments will be needed. For example, it would be of interest to analyze the expression of the key meristem development regulators in S65A- and S65D-complemented *cenh3-1* mutants.

The overall conclusion is that Aurora3 phosphorylates CENH3 at S65 and that this post-translational modification is required for the proper development of the floral meristem.

## Data Availability

All datasets generated for this study are included in the manuscript and/or the Supplementary Files.

## Author Contributions

DD, SH, TR, MK, and AH designed the study. DD, IL, SH, OW, TR, EDT, MK, PS, and CM conducted the experiments. DD, TR, MK, and AH analyzed the data. DD, IL, and AH drafted the manuscript.

## Conflict of Interest Statement

The authors declare that the research was conducted in the absence of any commercial or financial relationships that could be construed as a potential conflict of interest.

## Footnotes

1 www.finnzymes.com

2 www.dna-cloning.com

3 https://www.thermofisher.com

4 https://www.gelifesciences.com

5 www.lucigen.com

6 https://www.qiagen.com

7 https://www.jpt.com

8 www.merckmillipore.com

9 www.abcam.com

10 https://www.sigmaaldrich.com

11 https://www.neb.com

12 www.chromotek.com

13 https://www.clontech.com

14 http://www.dianova.de
