# Supplementary material for: Deregulated Phosphorylation of CENH3 at Ser65 Affects the Development of Floral Meristems in Arabidopsis thaliana
Source: Front Plant Sci. 2019 Jul 25;10:928. doi: 10.3389/fpls.2019.00928 (PMC6671561; doi:10.3389/fpls.2019.00928)
Supplement: Supplementary file 1 [file Data_Sheet_1.PDF]

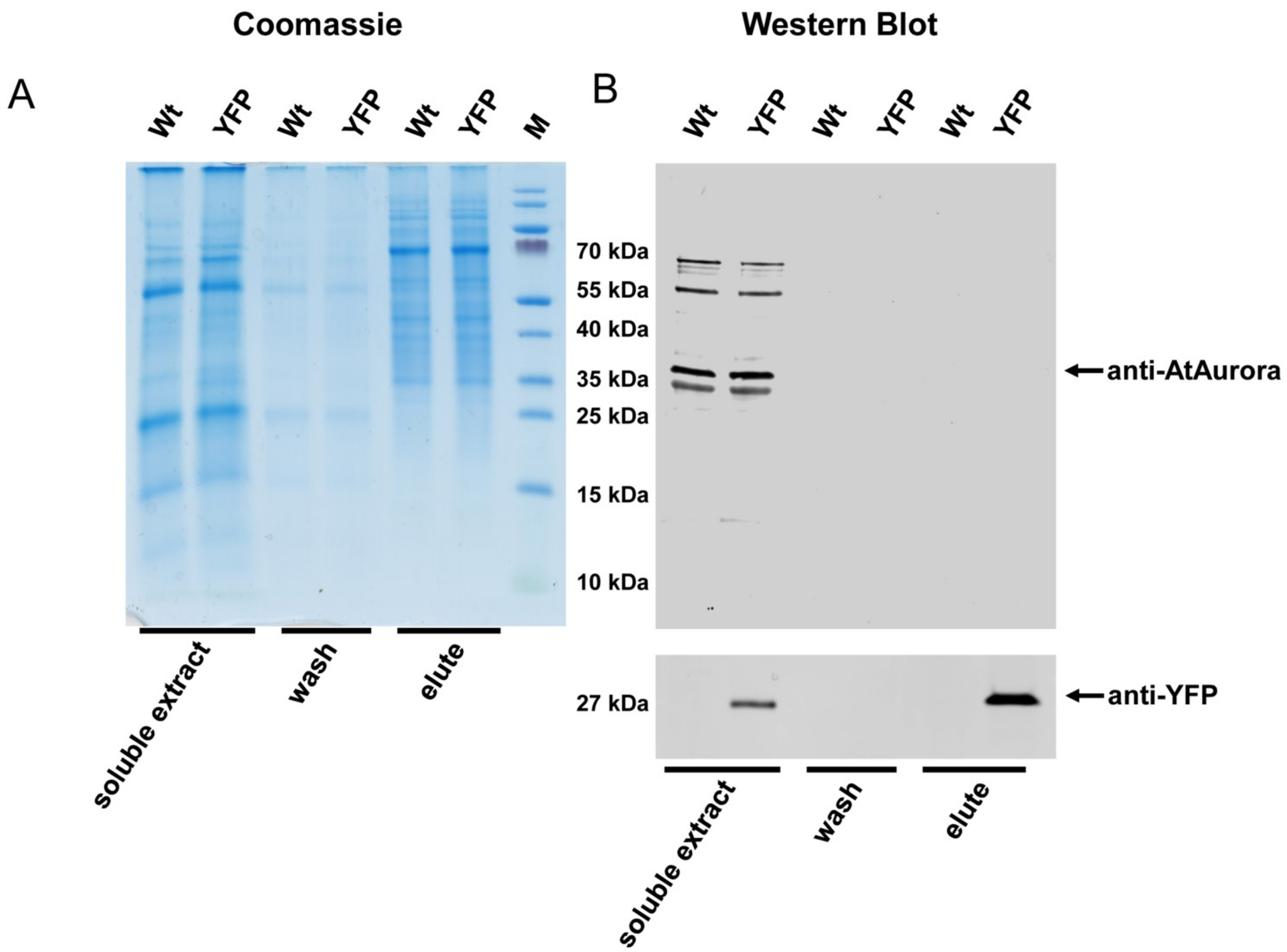

Figure S1. Negative control for the interaction of CENH3 with Aurora3 kinase of *A. thaliana*. (A, B) Western blot analysis using immuno-precipitated samples of *A. thaliana* EYFP transformants and Wt plants. (A) Proteins separated by gel electrophoresis and visualized by Coomassie Blue staining, (B) Western blot probed with an anti-AtAurora antibody. Protein extracted from Wt *A. thaliana* was used as additional control.

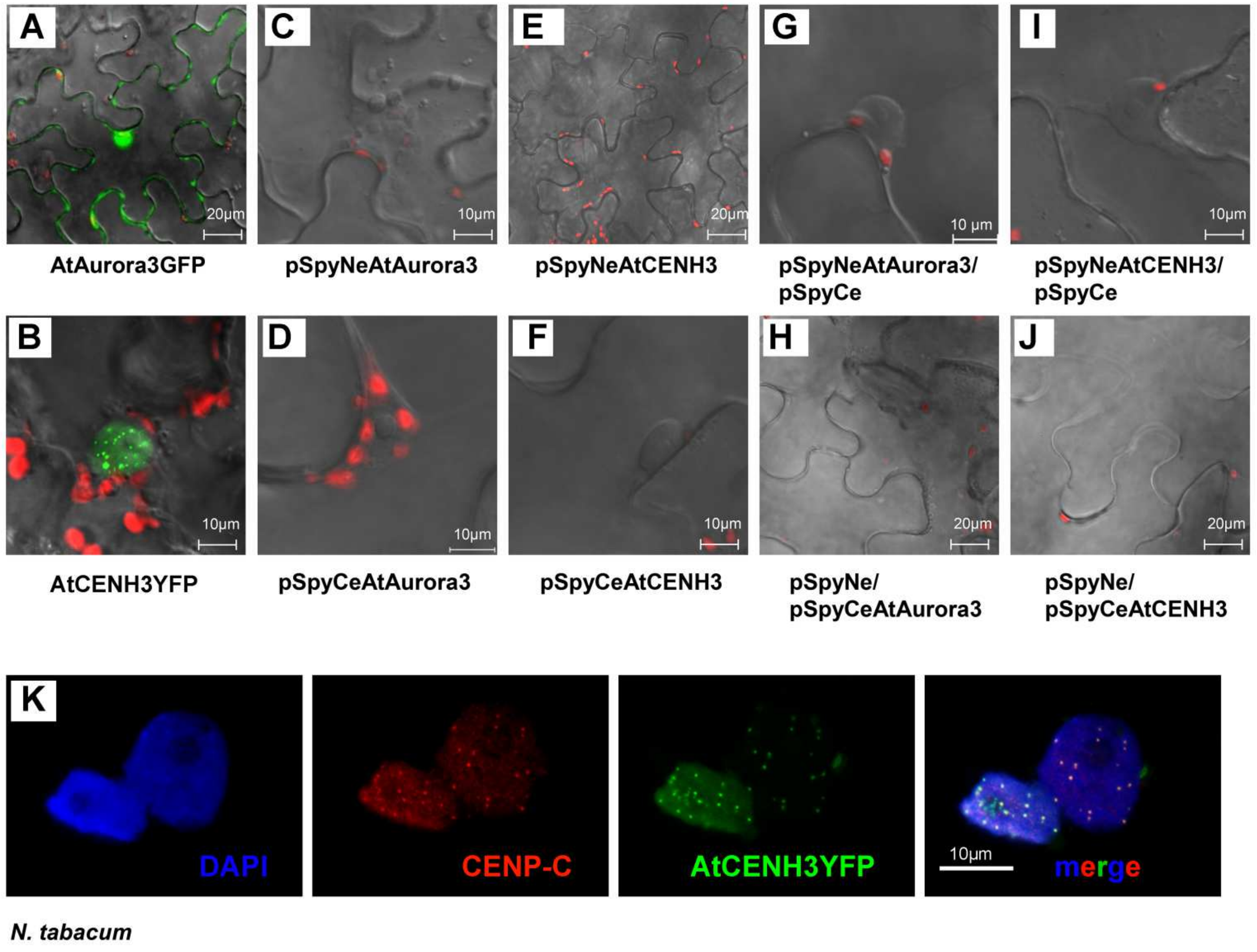

Figure S2. (A-J) Infiltration of *N. benthamiana* with *A. tumefaciens* harboring *Aurora3GFP* or *CENH3YFP* constructs. (A) Fluorescent signals indicative of *Aurora3* expression is visible in nuclei and at the cell periphery. (B) Centromere-like fluorescent signals indicative of *CENH3* expression is visible in only the nuclei. (C-G) Controls for the BiFC analysis of Aurora3 and CENH3: (C, D) Aurora3 and (E, F) CENH3. (G) pSpyNeAurora3/pSpyCe, (H) pSpyNe/pSpyCeAurora3, (I) pSpyNeCENH3/pSpyCe and (J) pSpyNe/pSpyCeCENH3. (K) The Centromere-like localization of *AtCENH3YFP* in transgenic *N. tabacum* visualized by double immunostaining using anti-YFP and anti-*Nicotiana* CENP-C antibodies.

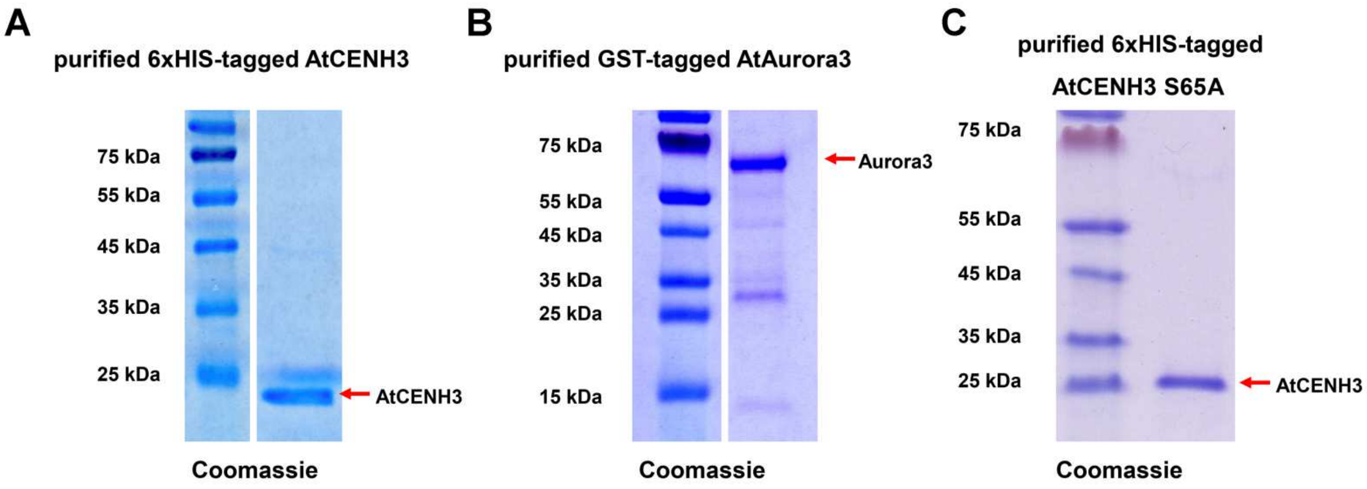

D

MARTKHRVTRSQPRNQTDAAGASSSQAAGPTTTPTRRGGEGGDNTQQTNPTTSPATGTRRGAKRS

RQAMPRGSQKKSYRYRPGTVALKEIRHFQKQTNLLIPAASFIREVRSITHMLAPPQINRWTAEALVALQ

EAAEDYLVGLFSDSMLCAIHARRVTLMRKDFELARRLGGKGRPW

E

Pep1: ARTKHRVTRSQPRNQTDAAG
Pep2: KHRVTRSQPRNQTDAAGA
Pep3: VTRSQPRNQTDAAGA
Pep4: RRGAKRSRQAMPRG
Pep5: SRQAMPRGSQKK
Pep6: SQKKSYRYRPG
Pep7: VALKEIRHFQKQTNLLIP
Pep8: NLLIPAASFIREVRSI
Pep9: IPAASFIREVRSITHMLAPP
Pep10: HMLAPPQINRWTAEALVALQ
Pep11: CAIHARRVTLMRKDFELARR

Figure S3. The heterologous expression of *A. thaliana* proteins in *E. coli.* (A) Coomassie stained Tris-glycine gel with CENH3, (B) Coomassie stained Tris-tricine gel with Aurora3, (C) Coomassie stained Tris-tricine gel with CENH3 S65A. The recombinant proteins were affinity-purified and used for a kinase assay. (D) Putative serine/threonine phosphorylation positions (in red) based on the Aurora kinase A/B recognition motif $(R/K)_{1\text{-}3}X(S/T)$. The N terminal region of CENH3 is shown in bold. (E) Peptides used for the *in vitro* kinase assay.

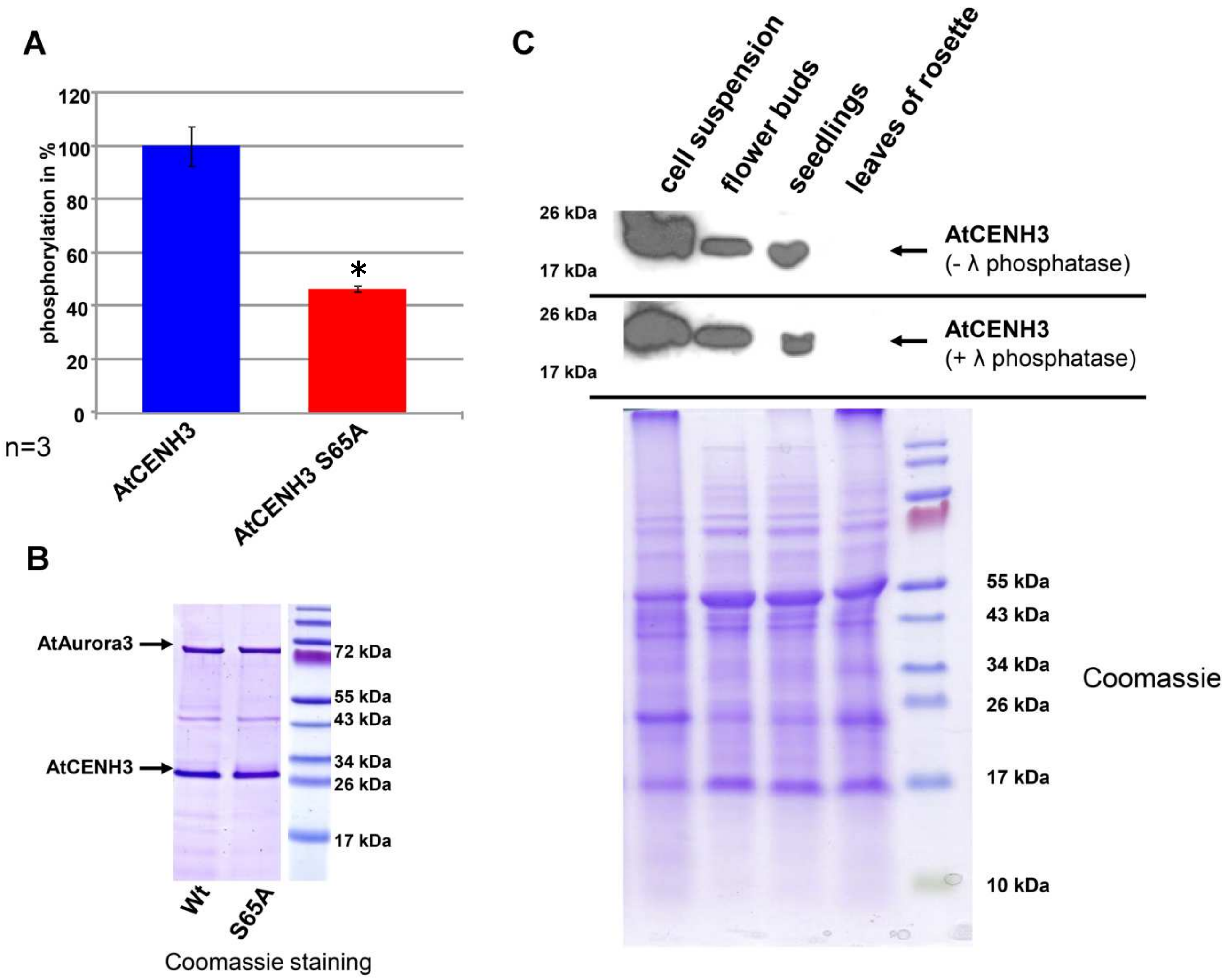

Figure S4. AtCENH3 mutated at the serine 65 residue is less efficiently phosphorylated by AtAurora3. (A) An average of phosphorylation efficiency in *in vitro* kinase assay of Aurora3 and recombinant CENH3 mutagenized at serine 65. Unmodified recombinant CENH3 was used as positive control. The experiment was performed in triplicates (technical repeats) and was analysed using LI-COR Image Studio 3.1 analytic software. Error bars correspond to standard deviation. AtCENH3 mutated at the serine 65 residue sample, indicated with asterisk is significantly different in comparison with Wt. The data were analyzed by one-way ANOVA-test ($*$=$p < 0.05$). The calculations were performed with the statistical program SigmaPlot v12 (Systat Software, Inc.). (B) Coomassie stained gel used as loading control for the recombinant proteins used in the kinase assay. (C) Western blot analysis based on antibodies raised against unmodified CENH3 used to quantify CENH3 in samples either exposed or not exposed to phosphatase treatment. Coomassie stained gels were used as the loading control.

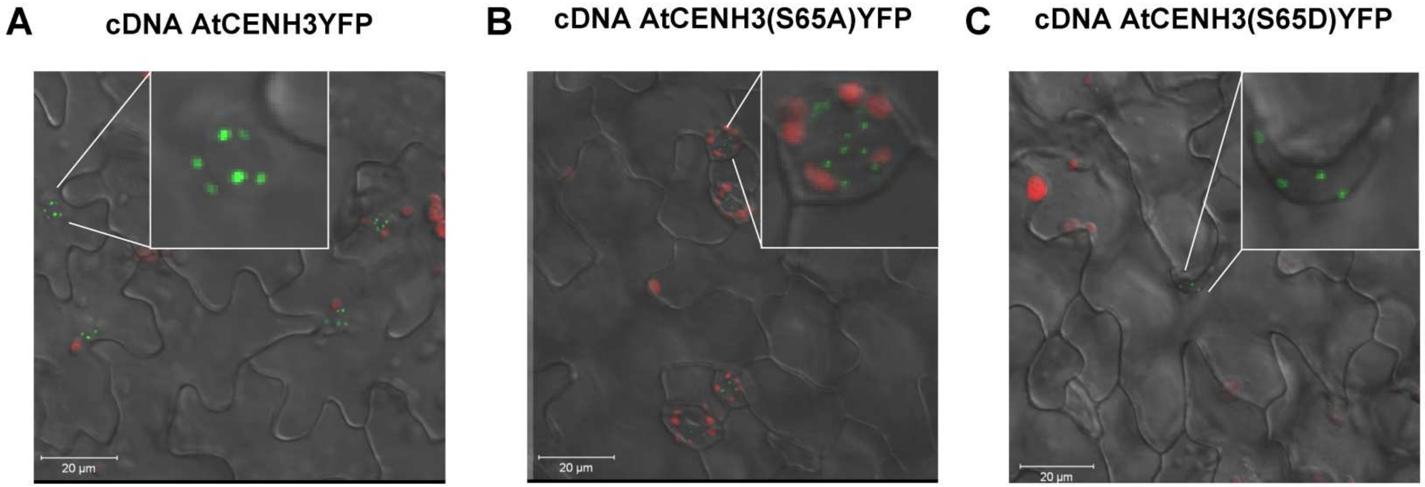

Figure S5. (A-C) Localization of AtCENH3 in *A. thaliana*. Centromere-like signals of p35S::*CENH3YFP* construct in *cenh3-1* transgenic plants (A) a unmutated *CENH3* transgene (17 independent lines), (B) *CENH3* S65A (11 independent lines), (C) *CENH3* S65D (19 independent lines).

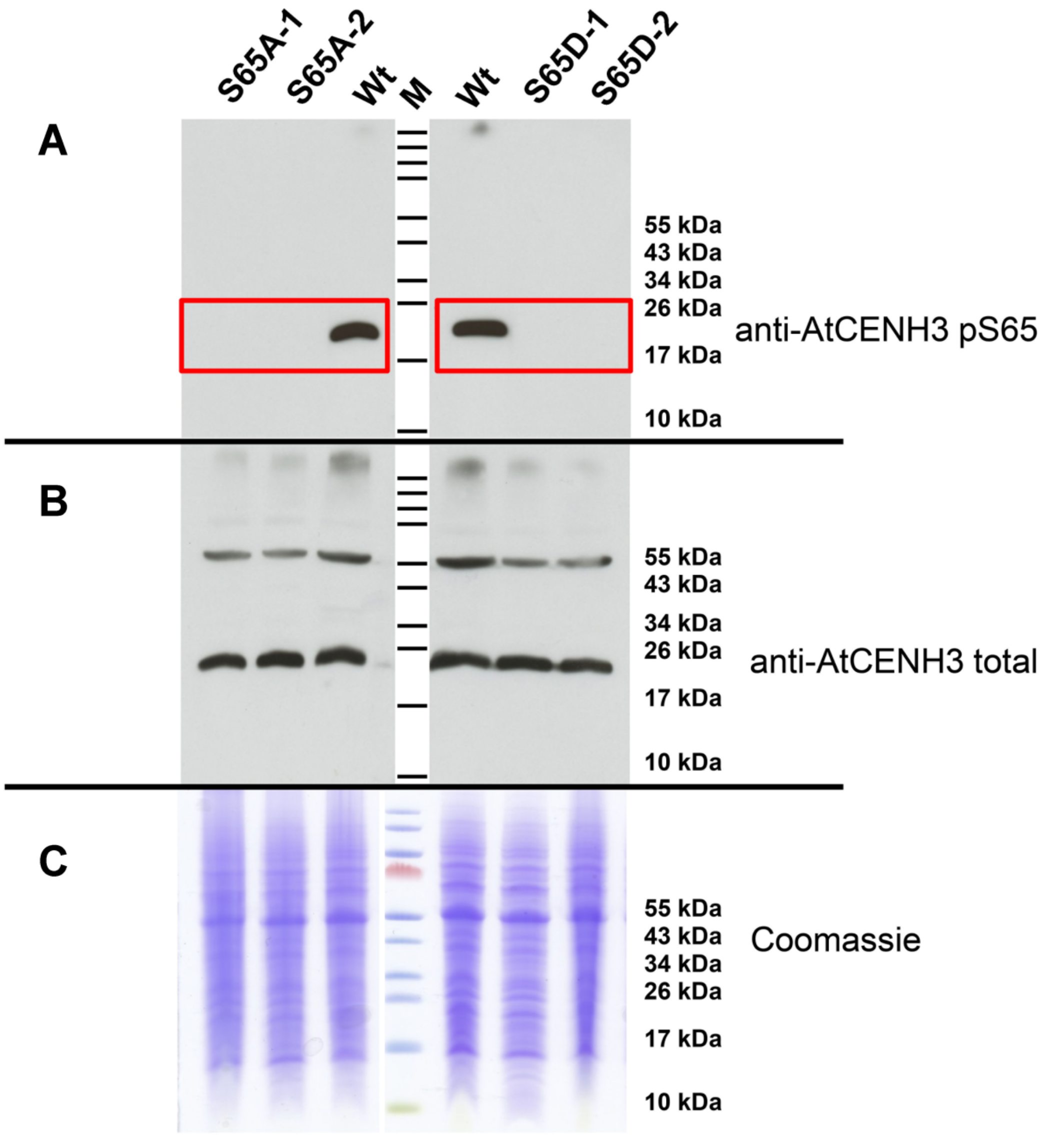

Figure S6. *A. thaliana cenh3-1* plants complemented with a mutated form of *AtCENH3* (S65A or S65D under native promotor) does not undergo phosphorylation at serine 65. (A) Western blot analysis of proteins isolated from *cenh3-1* mutant complemented with *CENH3* S65A or S65D and tested with anti-CENH3 pS65 antibodies. (B) Level of total CENH3 in *cenh3-1* mutants complemented by *CENH3* S65A or S65D. (C) Coomassie stained gels used as the loading control.

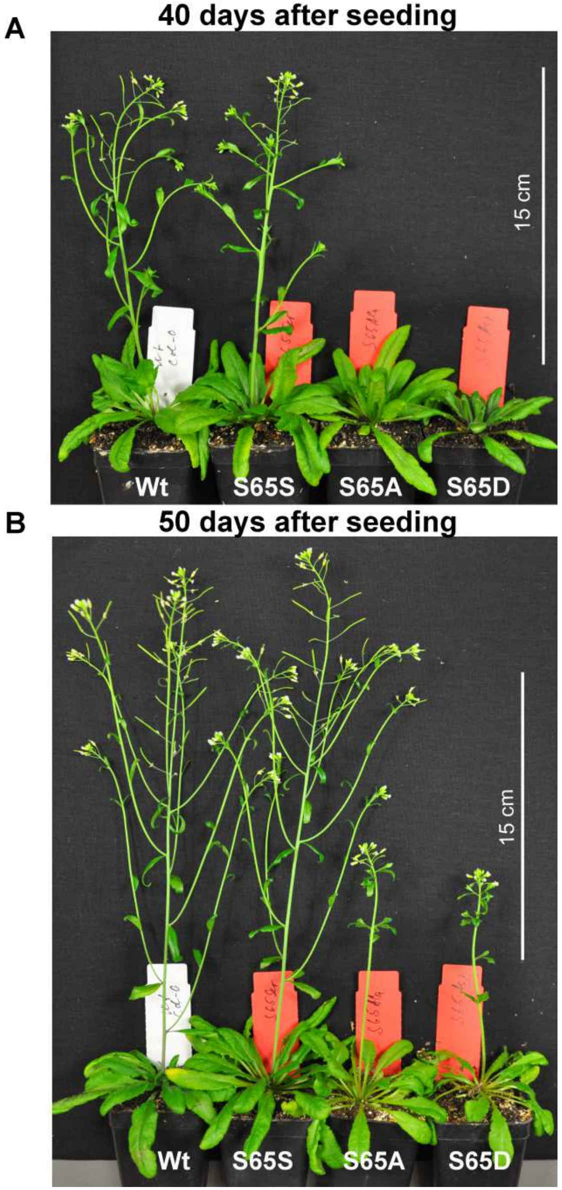

Figure S7. Differences in the developmental dynamics between *cenh3-1* complemented mutants (S65S, S65A or S65D under native promotor) and Wt. (A) 40 days and (B) 50 days after sowing.

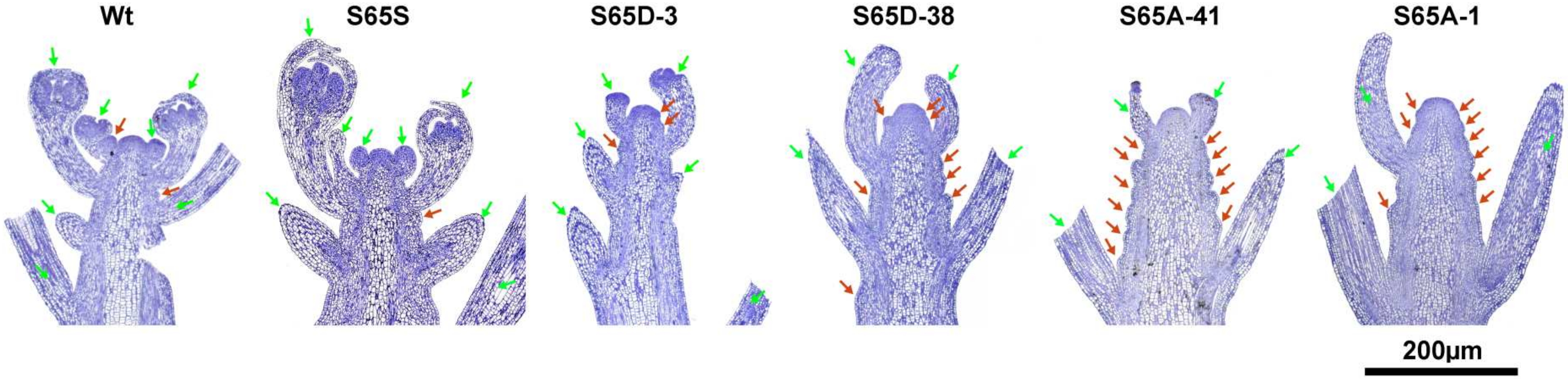

Figure S8. Light micrographs of developmental disorders of floral meristems of *cenh3-1* complemented mutants (S65S, S65A or S65D under native promotor) in comparison with Wt. Green arrows indicate normally developed floral meristems, brown arrows indicate undeveloped meristems.

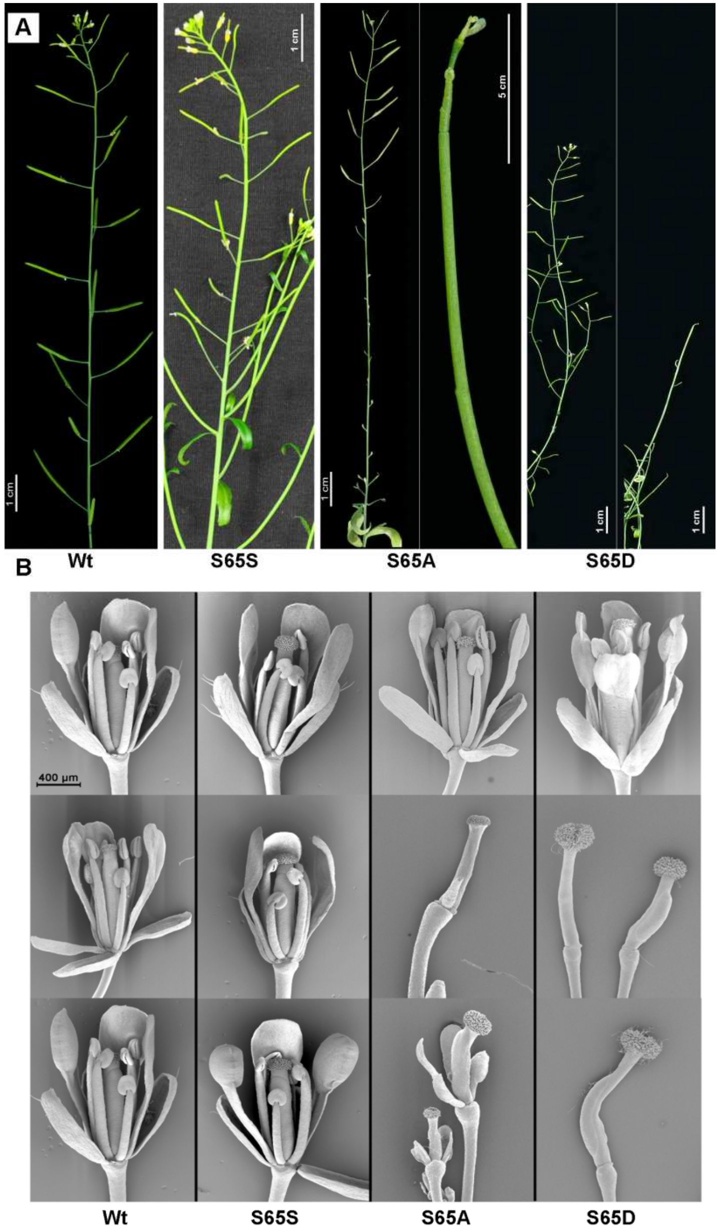

Figure S9. *cenh3-1* mutants complemented by *CENH3* S65A or S65D are defective with respect to their floral meristem development. (A) Main stems of Wt and *cenh3-1* complemented mutants (S65S, S65A or S65D under native promotor). (B) Scanning electron micrograph of flowers of Wt and *cenh3-1* complemented mutants (S65S, S65A or S65D under native promotor).

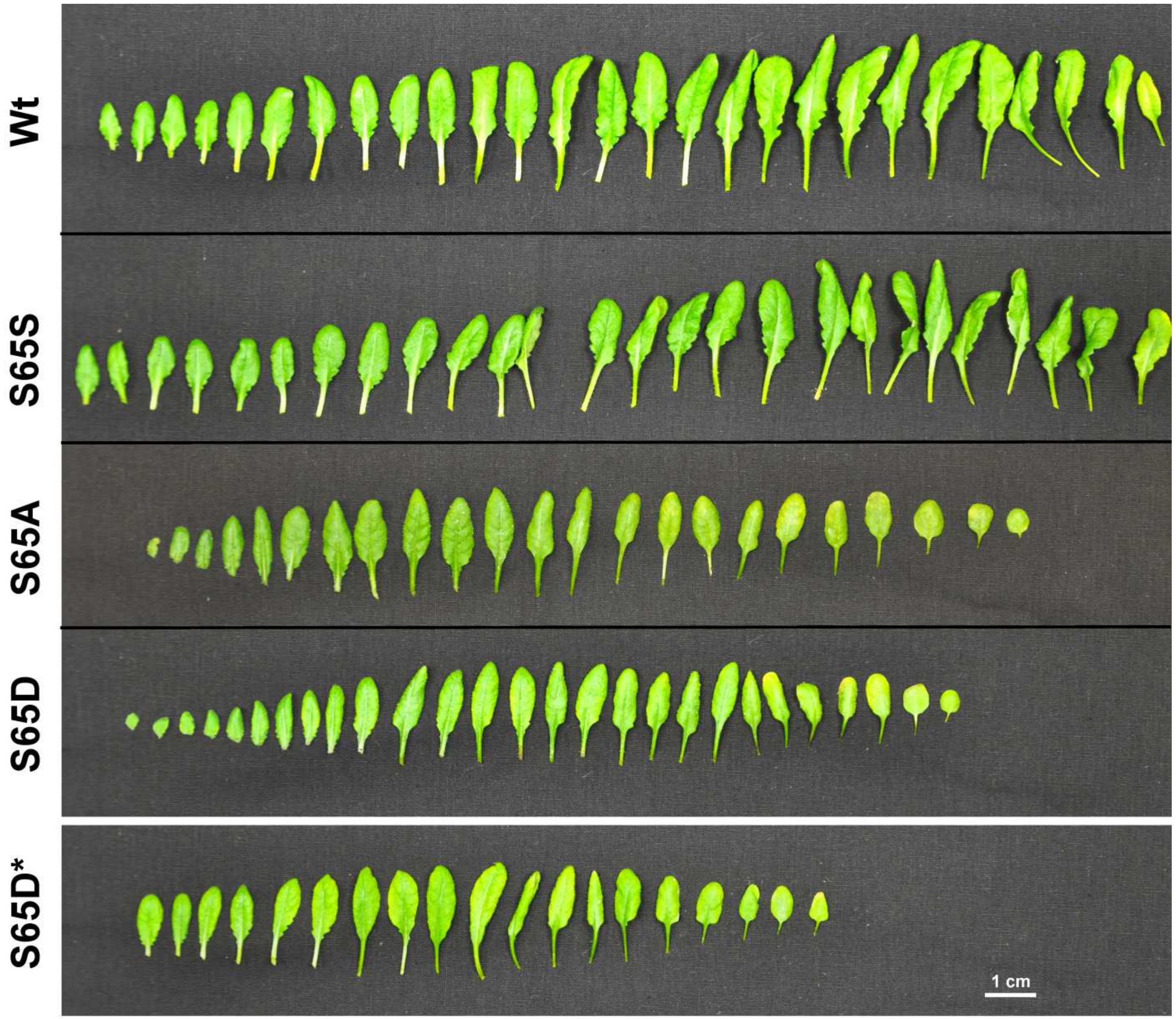

Figure S10. Phenotypical comparison of rosette leaves of Wt and *cenh3-1* complemented mutants (S65S, S65A or S65D under native promotor). Asterisk marks leaves of a plant with especially affected floral meristems.

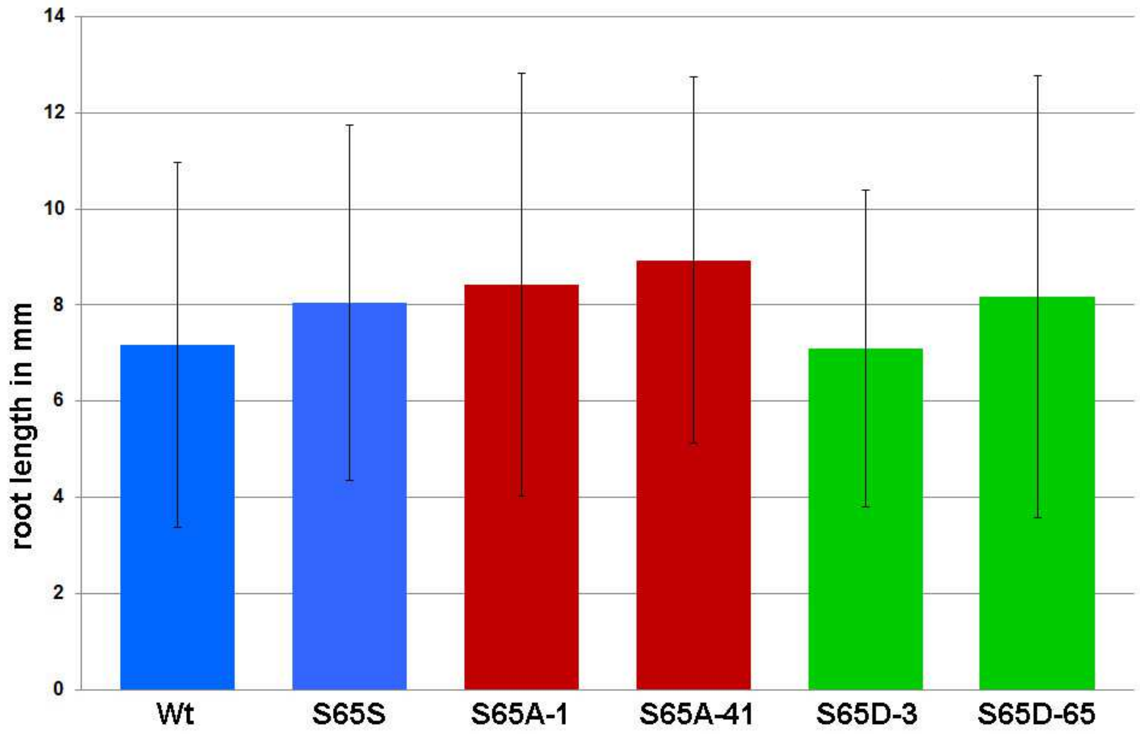

Figure S11. No significant differences in the average root length between Wt and *cenh3-1* complemented mutants (S65S, S65A or S65D under native promotor) were detected. Root length was measured 5 days after seeding (DAS). For Wt and S65S, size of 50-seedling roots was measured. For S65A and S65D constructs 2 independent transgenic lines were analysed (for each 50 seedlings). Error bars correspond to standard deviation.

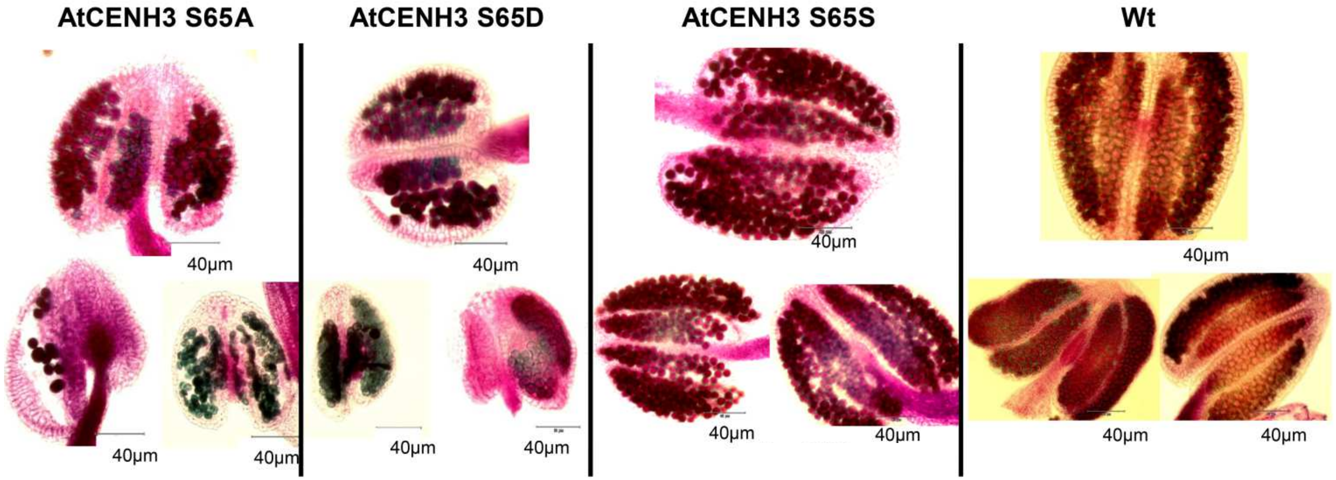

Figure S12. Pollen produced by a *cenh3-1* mutant complemented by *CENH3* mutated at serine 65. Wt and *cenh3-1* mutant complemented with a Wt copy of *CENH3* were used as the control. At least fifteen anthers per plant, three plant per independent line and seven independent line were analysed for each construct.

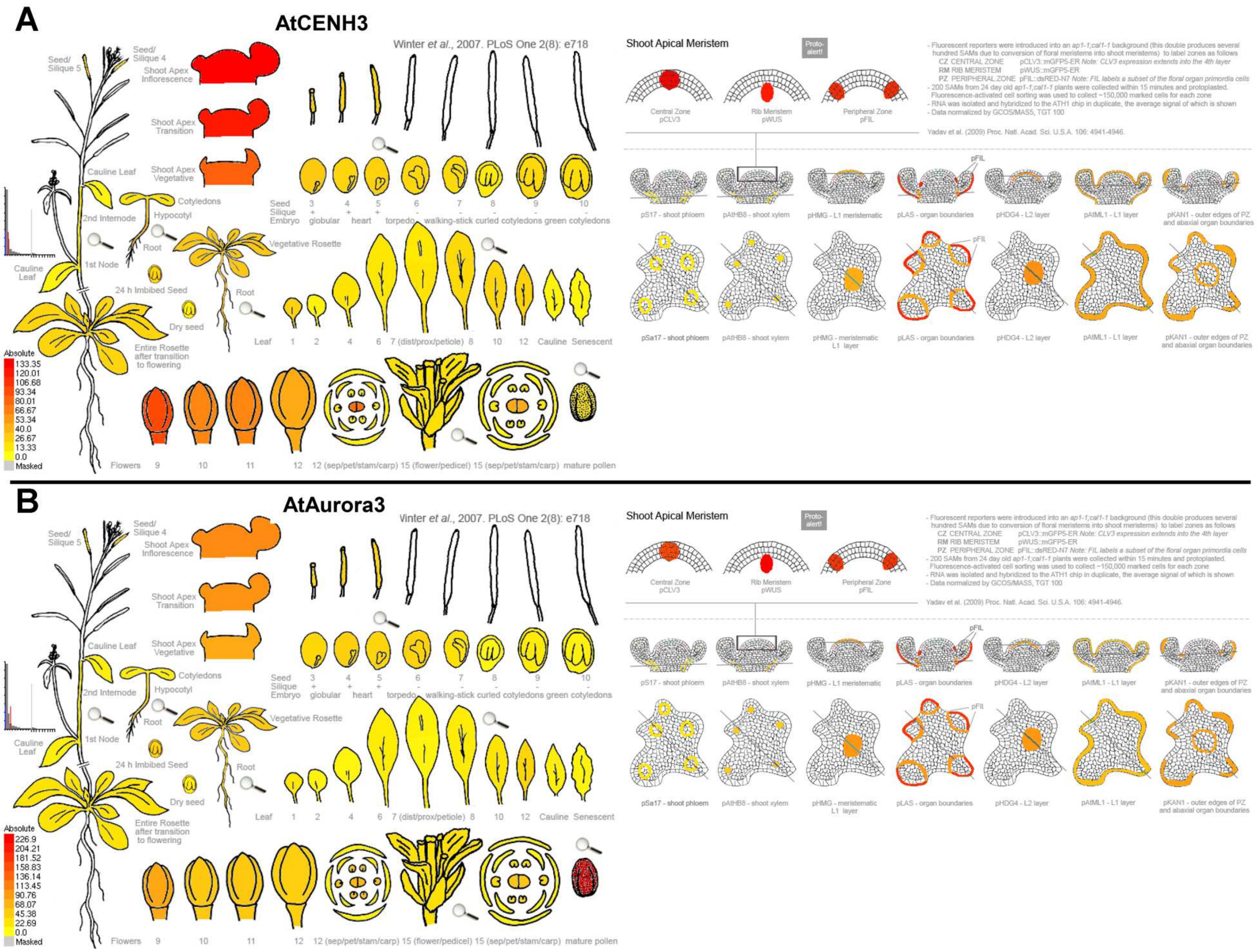

Figure S13. The transcription profiles of (A) *CENH3* and (B) *Aurora3* in *A. thaliana* according to the *A. thaliana* eFP Browser (bar.utoronto.ca/efp/cgi-bin/efpWeb.cgi). The abundance of transcripts is similar for both genes.

A

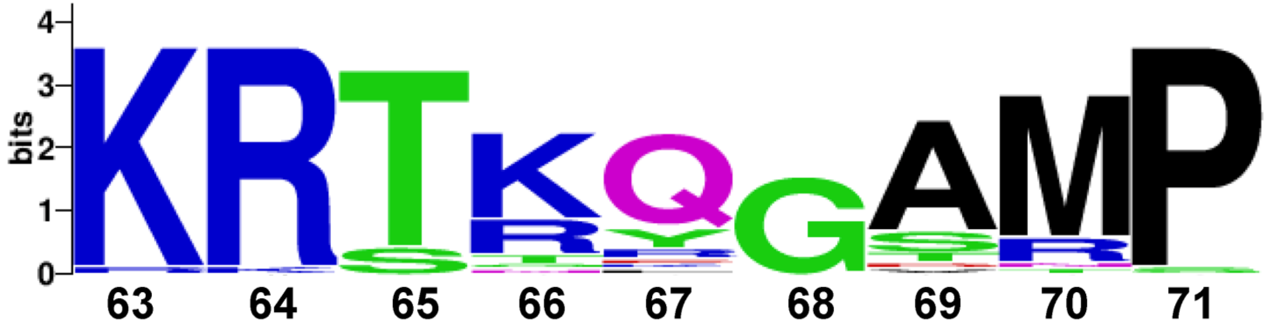

B

definition/plant species	gene bank number	position/sequence	
centromeric protein HTR12 [Arabidopsis thaliana]	AAM64429	59	KRSRQ-AMP
centromeric histone H3-like protein-2 [Cardamine flexuosa]	BAF49722	56	KRSRQ-AMP
centromeric histone H3-like protein-3 [Cardamine flexuosa]	BAF49723	56	KRSRQ-AMP
centromeric histone H3-like protein-1 [Cardamine flexuosa]	BAF49721	57	KRTRR-AMP
hypothetical protein EUTSA_v10008889mg [Eutrema salsugineum]	XP_006418417	61	KRTNQ-AMP
centromeric histone H3-like protein-4 [Cardamine flexuosa]	BAF49724	53	KRTRR-DMP
centromeric histone H3-like protein_1 [Arabis hirsuta]	BAF49719	56	KRT----MP
centromeric histone H3-like protein_1 [Lepidium virginicum]	BAF49731	61	KRTKE-TMP
centromeric histone H3 protein [Orychophragmus violaceus]	ALK04338	60	KRTKQ-ANP
CENH3 [Lepidium oleraceum]	AJF96734	61	KRTGK-VMP
centromeric protein HTR12 [Camelina sativa]	XP_010474733	58	KRTRYgSRS
centromeric protein HTR12 [Camelina sativa]	XP_010457134	59	KRTKYgSRP
centromeric protein HTR12 isoform X1 [Camelina sativa]	XP_010480342	48	KRTKYgSRP
centromeric protein HTR12 isoform X2 [Tarenaya hassleriana]	XP_010556592	120	RKSRLgTTP
centromeric histone H3-like protein_1 [Raphanus sativus]	BAF49733	60	KRTTQ-AMP
centromeric histone H3-like protein_2 [Raphanus sativus]	BAF49734	61	KRTTQ-AMP
centromeric histone H3-like protein [Brassica oleracea var. italica]	BAF63142	64	KRTKQ-AMP
centromeric protein HTR12 isoform X2 [Brassica oleracea var. oleracea]	XP_013602731	65	KRTKQ-AMP
centromeric protein HTR12 isoform X2 [Brassica napus]	XP_013677169	62	KRTKQ-AMP
centromeric protein HTR12 isoform X1 [Brassica oleracea var. oleracea]	XP_013602730	66	KRTKQ-AMP
centromeric protein HTR12 isoform X2 [Brassica napus]	XP_013670392	60	KRTKQ-AMP
centromere-specific H3 variant protein [Brassica juncea]	ACZ04980	62	KRTKQ-AMP
centromeric protein HTR12 isoform X1 [Brassica napus]	XP_013670391	61	KRTKQ-AMP
centromeric protein HTR12 isoform X1 [Brassica rapa]	XP_009118807	63	KRTKQ-AMP
centromeric histone H3-like protein-2 [Brassica juncea]	BAF49729	58	KRTKQ-AMP
centromeric histone H3-2 protein [Brassica juncea]	ALK04339	59	KRTKQ-AMP
centromeric protein HTR12 isoform X3 [Brassica rapa]	XP_009118809	61	KRTKQ-AMP
centromeric protein HTR12 [Brassica rapa]	NP_001288957	62	KRTKQ-AMP
centromeric protein HTR12 isoform X2 [Camelina sativa]	XP_010480356	29	KRTKYgSRP
centromeric histone Opu1HTR12 [Olimarabidopsis pumila]	AAT96389	58	KRARQ-AMP
centromeric histone H3 HTR12 [Arabidopsis arenosa]	AAL86777	61	RRARQ-AMP

Figure S14. Part of the N- terminal region of CENH3 is present in most *Brassicaceae* species. (A) Frequency of amino acids in the region surrounding serine 65 of CENH3. (B) Sequence alignment of *Brassicaceae* CENH3.

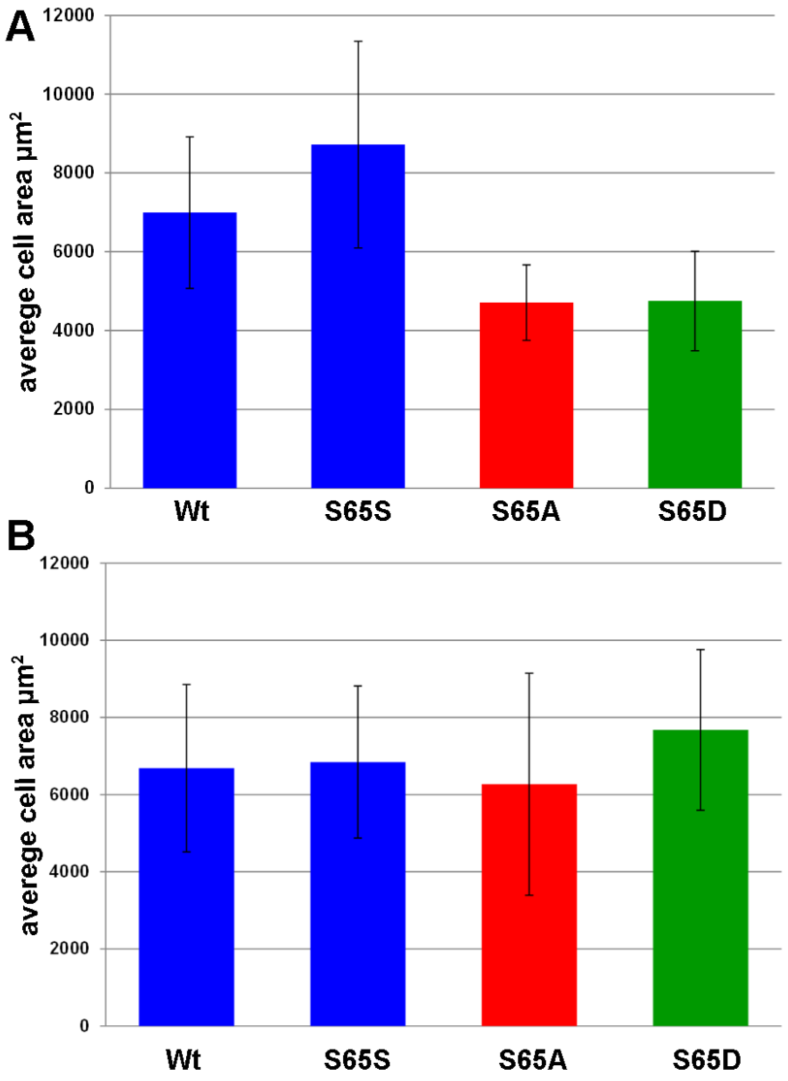

Figure S15. Average cell area in the leaf abaxial epidermis of *cenh3-1* complemented mutants (S65S, S65A and S65D under native promotor) and Wt in μm^2. Cell area was analysed using Zeiss ZEN2012 SP4 analytic software. Error bars correspond to standard deviation. (A) - 30 days and (B) - 50 days after sowing. 7 plants were analysed for control and each CENH3 mutated variants. For each CENH3 mutated variant 5 independent transgenic lines were included.

Table S1. Sequences of primers used.

Primer	Sequence
**Primers to mutate S65 (gDNA)**	
S65_A_for	GGCTAAGAGAGCTAGACAGGCTATG
S65_D_for	GGCTAAGAGAGACAGACAGGCTATG
S65_A+D_gDNA_rev	CCTCTCTTGACAAACCAAAACGAG
**Primers to mutate S65 (cDNA)**	
S65_A+D_cDNA_rev	CCTCTCCTTGTACCAGTAGCTGGTG
**Mutants specific primers**	
cenh3-1_mut_for	GGTGCGATTTCTCCAGCAGTAAAAATC
cenh3-1_mut_rev	CTGAGAAGATGAAGCACCGGCGATAT
cenh3-1_mut2429r	AACTTTTGCCATCCTCGTTTCTGTT
**Primers for Gateway cloning**	
AtAurora3G-LP	CACCATGAGTAAGAAATCGACAGAATCTGACGCTG
AtAurora3G-RP	AATATCAATTGAGGCACACACACCTTTCGGATC
**Recombinant proteins expression**	
CENH3_expr_for	ATGGCGAGAACCAAGCATCGCGTTAC
CENH3_expr_rev	CCATGGTCTGCCTTTTCCTCCAAGC

Table S2. Chronological growth stages of *cenh3-1* complemented mutants (S65S, S65A or S65D) and Wt plants in days after seeding (DAS). For each mutated variant seven independent transgenic lines were analysed. The total number of plants analysed for control and each mutated variant was 42.

lines	germination (DAS)	leaf production and rosette growth (DAS)	flowering (DAS)	silique ripening (DAS)
Wt	1-7	8-35	32-51	48-63
S65S	1-7	8-36	33-52	47-64
S65D	1-7	8-46	42-63	56-74
S65A	1-7	8-47	44-62	58-76

Table S3. Phenotypical analysis of *cenh3-1* complemented mutants (S65S, S65A and S65D) and Wt plants. The number of leaves and lateral stems at different developmental stages were counted. For control and each CENH3 mutated variant 21 plants were analysed. For each CENH3 mutated variant 7 independent transgenic lines were included (SD - standard deviation). Reduced number of lateral stems was observed for 92% of the S65D- and 93% of the S65A- complemented plants 50 DAS. Data values indicated with asterisks were significantly different in comparison with Wt. The data were analyzed by one-way ANOVA-test ($*=p < 0.05$). The calculations were performed with the statistical program SigmaPlot v12 (Systat Software, Inc.).

lines	number of leaves (30 DAS)		number of lateral stems (50 DAS)		number of lateral stems (63 DAS)	
	average	SD	average	SD	average	SD
Wt	26.40	1.43	8.10	1.66	9.50	1.27
S65S	25.60	1.26	7.60	1.26	10.20	1.48
S65D	24.60 *	1.35	3.60 *	1.26	13.40 *	1.58
S65A	26.10	1.20	3.50 *	0.97	13.30 *	1.34
